# An Analysis of the Role of the Indigenous Microbiota in Cholesterol Gallstone Pathogenesis

**DOI:** 10.1371/journal.pone.0070657

**Published:** 2013-07-29

**Authors:** Jacqueline J. Fremont-Rahl, Zhongming Ge, Carlos Umana, Mark T. Whary, Nancy S. Taylor, Sureshkumar Muthupalani, Martin C. Carey, James G. Fox, Kirk J. Maurer

**Affiliations:** 1 Division of Comparative Medicine, Massachusetts Institute of Technology, Cambridge, Massachusetts, United States of America; 2 Division of Gastroenterology, Brigham and Women’s Hospital and Department of Medicine, Harvard Medical School, Boston, Massachusetts, United States of America; 3 Department of Biological Engineering, Massachusetts Institute of Technology, Cambridge, Massachusetts, United States of America; 4 Department of Biomedical Sciences and Center for Animal Resources and Education, Cornell University, Ithaca, New York, United States of America; University of Aberdeen, United Kingdom

## Abstract

**Background and Aims:**

Cholesterol gallstone disease is a complex process involving both genetic and environmental variables. No information exists regarding what role if any the indigenous gastrointestinal microbiota may play in cholesterol gallstone pathogenesis and whether variations in the microbiota can alter cholesterol gallstone prevalence rates.

**Methods:**

Genetically related substrains (BALB/cJ and BALB/cJBomTac) and (BALB/AnNTac and BALB/cByJ) of mice obtained from different vendors were compared for cholesterol gallstone prevalence after being fed a lithogenic diet for 8 weeks. The indigenous microbiome was altered in these substrains by oral gavage of fecal slurries as adults, by cross-fostering to mice with divergent flora at <1day of age or by rederiving into a germ-free state.

**Results:**

Alterations in the indigenous microbiome altered significantly the accumulation of mucin gel and normalized gallbladder weight but did not alter cholesterol gallstone susceptibility in conventionally housed SPF mice. Germ-free rederivation rendered mice more susceptible to cholesterol gallstone formation. This susceptibility appeared to be largely due to alterations in gallbladder size and gallbladder wall inflammation. Colonization of germ-free mice with members of altered Schaedler flora normalized the gallstone phenotype to a level similar to conventionally housed mice.

**Conclusions:**

These data demonstrate that alterations in the gastrointestinal microbiome may alter aspects of cholesterol gallstone pathogenesis and that in the appropriate circumstances these changes may impact cholesterol cholelithogenesis.

## Introduction

The prevalence of cholesterol gallstones has increased in recent years, especially in the Western world where contributing factors include diet and subsequent obesity [1]. The disease is of concern due to its clinical and economic significance. Clinically, gallstones can induce cholecystitis, cholangitis, acute pancreatitis, and promote biliary cancer [2]. Because there are currently no effective preventative or nonsurgical treatments, surgical intervention to remove gallstones and the gallbladder is necessary in symptomatic cases resulting in a high annual healthcare burden in the United States [3].

Both genetic and environmental factors contribute to the formation of cholesterol gallstones. In a study analyzing 43,141 twin pairs the approximate phenotypic contribution to symptomatic gallstones was 25% for genetics, 13% for shared environmental factors and 62% for unique environmental factors [4]. With regard to genetic factors, the disease is almost invariably polygenic likely involving genes of cholesterol transport and metabolism [5]. Recently we demonstrated in inbred mice that immune function genes specifically genes important in adaptive immunity are also important genetic determinants [6]. Environmental factors that are thought to or known to promote cholesterol gallstones include estrogen and progestogens, cholesterol-lowering medications, obesity and rapid weight loss [7], [8]. More recently, our group demonstrated that in some strains of mice infection with some enterohepatic *Helicobacter* spp. can promote cholesterol gallstones [9]. These data demonstrate microbes may influence host gallstone phenotype.

In previous studies, we observed a lower prevalence of cholesterol gallstones in BALB/c mice from Taconic Farms (Hudson, NY, BALB/cAnNTac) in comparison to BALB/c mice from The Jackson Laboratory (Bar Harbor, ME, BALB/cJ) [6]. We hypothesized that these differences might be due to the markedly different husbandry conditions between these two institutions. Taconic Farms rederives their mice into a germ-free state and then colonizes them with altered Schaedler Flora (ASF; a group of 8 known commensal bacterial species) and then maintains them under specific-pathogen free (SPF) conditions [10]. Mice from the Jackson Laboratory are not rederived into a germ-free state, but instead are maintained under SPF conditions. A similar finding was demonstrated when mice from Taconic Farms were found to be less susceptible to infection with *Giardia lamblia* in comparison to mice from the Jackson Laboratory [11]. Subsequent prospective studies involving fecal gavage and co-housing of strains proved that these differences in enteric disease susceptibility were attributable to differences in the gastrointestinal microbiota [11]. More recent studies in both humans and mice clearly demonstrate that the intestinal microbiome alters proclivity to a variety of metabolic diseases including obesity and diabetes [12]–[14]. Further, since we have demonstrated a role of the immune system in gallstone pathogenesis, microbes may exert an influence by modulating the immune response [6].

The observable difference in cholesterol gallstone phenotype between substrains of BALB/c mice may also be attributed to genetic differences between the mice. Between 1920 and 1970, approximately 18 substrains of BALB/c mice were developed [15]. Analysis of genetic markers between the substrains demonstrated that BALB/c mice have diverged mostly through spontaneous mutation and residual heterozygosity, with known differences in the *Raf1* locus (controls expression of alpha-fetoprotein), the *Qa2* locus (controls cell surface antigens), the *Gdc1* locus (controls L-glycerol 3-phosphate dehydrogenase activity in the liver) and the PR1 repetitive sequence [16]. However, in a detailed analysis of substrains there were only 4 of 2000 single nucleotide polymorphisms (SNPs) which differed between BALB/cJ and BABL/cByJ substrains indicating that BALB/c substrains are still genetically highly related (99.8% identical) [17].

In this study, we analyzed the relative contributions of the indigenous gastrointestinal microbiota and genetic differences in closely related BALB/c mouse strains in diet-induced cholesterol gallstone formation. We asked whether the indigenous gastrointestinal microbiota or small genetic differences were responsible for the marked differences observed among these closely related substrains. We compared cholesterol gallstone prevalence rates and employed quantitative PCR of mouse ceca to determine the relative abundance of altered Schaedler flora (ASF) which we used as a marker of microbial transfer [18]. Crossfaunation (transfer of bacteria to mice with established microbiota) and crossfostering (transfer of bacteria to mice without an established microbiota) as a means of altering the microbiome was utilized to determine whether cholesterol gallstone prevalence would be altered under conditions of specific pathogen free housing (SPF). Finally, we re-derived mice into a germ-free state and housed them in germ-free isolators. We then compared their gallstone phenotype to mice that were housed under SPF status or those that were re-derived and colonized with known microbes and housed in germ-free microisolators. These studies were undertaken to begin to elucidate the relative contribution of the intestinal microbiome utilizing mice which are highly identical genetically but which display markedly different cholesterol gallstone phenotypes. The data presented herein provide evidence that differences in the host microbiome alters components of the cholesterol gallstone phenotype and that microbial modulation can alter the cholesterol gallstone phenotype of mice primarily by altering gallbladder motility and inflammation.

## Materials and Methods

### Ethics Statement

Mice were maintained in an Association for Assessment and Accreditation of Laboratory Animal Care International (AAALAC) accredited facility. All animal work was done in accordance with PHS guidelines and was approved by MIT’s institutional animal care and use committee (Protocol # 0108-008-11).

### Specific Pathogen Free Studies Including Substrain Experiments, Cross-faunation and Cross-fostering Studies

Mice were housed in microisolator cages under specific pathogen free conditions (free of known pathogenic viruses, bacteria and fungi). They were provided with Prolab 3000 (Purina Mills, St. Louis, MO) prior to study and then with a synthetic lithogenic diet (containing 1% cholesterol, 0.5% cholic acid, and 15% dairy derived triglyceride; Jackson laboratories Bar Harbor, ME) and provided water ad libitum. Mice of various microbial groups were housed in cubicles or on independent rack systems and personal protective equipment were changed in between handling various groups to minimize cross-colonization with microbes. Mice were procured fromeither The Jackson Laboratory (BALB/cJ and BALB/cByJ strain; Bar Harbor, ME; referred to as Jackson) or Taconic Farms, Inc. (BALB/cAnNTac and BALB/cBomTac Germantown, NY; referred to as Taconic). For all SPF studies male 6–8 week old BALB/cJ and BALB/cAnNTac served as controls (n = 15–25) and represented “susceptible” and “resistant” strains respectively. Studies were conducted simultaneously or in close proximity and data from smaller cohorts of controls were combined to reach n = 15–25 control animals.

### Substrain Experiments

Closely related substrains of mice housed at different vendor sources were compared to determine if there were differences in gallstone phenotype. BALB/cJ and BALB/cJBomTac mice are derived from the same genetic substrain, but are produced and housed at their respective vendors. Likewise, BALB/cByJ and BALB/cAnNTac are derived from the same original substrain but produced, housed and maintained at Jackson and Taconic respectively (Figure S1) [15]. At 8 weeks of age male BALB/cJBomTac (n = 15), BALB/cByJ (n = 19) and controls were fed a lithogenic diet for 8 weeks.

### Crossfaunation Experiments

To determine if modifications in the already established indigenous gastrointestinal microbiota of mice alters cholesterol gallstone phenotype, adult crossfaunation experiments were performed. Male, 6-week-old, BALB/cJ mice and BALB/cAnNTac mice were studied. Feces were collected from these mice. Five fecal pellets from each group were placed in 1 ml of thioglycolate and homogenized. Samples were centrifuged until the solid debris and supernatant were separated. Each experimental mouse was orally gavaged with 0.2 ml of the supernatant such that BALB/cAnNTac mice (n = 10) was gavaged with fecal supernatant from BALB/cJ mice and BALB/cJ mice (n = 10) was gavaged with fecal supernatant from the BALB/cAnNTac mice (Figure S2). Crossfaunated mice were co-housed for 2 weeks to ensure fecal bacterial sharing between the two groups. At 8 weeks of age, all mice were converted to a lithogenic diet and fed the diet for 8 weeks. Mice were maintained in co-housed units throughout the study. Data were compared to previously described unmanipulated BALB/cJ and BALB/cAnTac mice.

### Crossfostering Experiments

To determine if modifications in the indigenous gastrointestinal microbiota of mice prior to establishment of a resident microbiota would alter cholesterol gallstone phenotype, neonatal crossfostering experiments were performed. Within 24-hours of birth, pups from BALB/cAnNTac females were cross-fostered to BALB/cJ females and BALB/cJ pups were cross-fostered to BALB/cAnNTac females (Figure S3). Crossfostered pups were then bred to one another and 8 week old male progeny from these crosses were utilized for future experiments. At 8 weeks of age, male progeny (n = 17–18) were fed a lithogenic diet for 8 weeks. Data were compared to previously described lithogenic diet fed BALB/cJ and BALB/cAnTac mice.

### Germ-Free Experiments

BALB/cByJ mice were purchased and rederived into a germ-free state by embryo-transfer into germ-free recipient CD-1 mice. Mice were maintained in germ-free flexible-film isolators and monitored weekly for germ-free status by environmental culture as described previously [19], [20]. A cohort of rederived germ-free BALB/cByJ mice was placed into another germ-free isolator and these mice were dosed orally and rectally with freeze-thawed cultured individual stocks of ASF (Taconic Farms, Germantown, NY). Progeny of mice inoculated with these agents were used for subsequent studies. ASF colonization was confirmed by cecal quantitative PCR of adult mice (n = 5 per group; Figure S4). At 8-weeks-old, male progeny of germ-free and gnotobiotic ASF mice (n = 12 each) were fed an irradiated vacuum-sealed, lithogenic diet containing 1% cholesterol, 0.5% cholic acid and 15% triglyceride (Harlan-Teklad, TD.09237) for 8 weeks. An additional group of male (n = 12) conventional BALB/cByJ mice were fed this diet and were housed under standard SPF conditions as described previously. Gallbladder lipid analysis was performed on a male germ-free and conventional mice (n = 5 each) fed this diet as previously described [21].

### Bile Analysis, Tissue Analysis, and Histopathologic Evaluation

Mice were fasted for approximately 12 hours before being euthanized via CO_2_ asphyxiation followed by necropsy. Gallbladders were removed intact, weighed, and bile was removed for biliary phenotype analysis by direct and polarized light microscopy. Normalized gallbladder weight was determined by the weight of the gallbladder (mg) over the weight of the mouse (g). The mucin gel score (% mucin in bile) was assessed on a 0–5 scoring scale (0 = 0% mucin in the bile, 1 = 20% of gallbladder bile volume consisted of mucin, 2 = 40% of bile consisted of mucin, 3 = 60% of bile consisted of mucin, 4 = 80% of bile consisted of mucin, 5 = 100% of bile consisted of mucin) [6], [21]. Bile was also assessed by polarized light microscopy for the percent prevalence of liquid crystals, solid crystals, sandy stones, and cholesterol gallstones the temporal sequence in which cholesterol gallstones form in gallbladder bile of the mouse. In rare instances gallbladders were not harvested completely intact precluding complete phenotypic analysis. Empty gallbladders, were either immediately flash frozen in liquid N_2_ and stored at −80°C for gene expression analysis or were fixed in 10% neutral buffered formalin for histopathological analysis. In addition, ceca were also flash frozen and stored at −80°C for quantitative PCR analysis.

### Cecal DNA Extraction and Quantitative PCR of ASF Species

To quantify ASF colonization in mouse gastrointestinal contents, cecal DNA was extracted from a subset of mice (n = 5 per experimental group) using the All-Prep DNA/RNA Mini Kit (Qiagen, Valencia, CA). Primer sequences (Integrated DNA Technologies, San Diego, CA) for the 8 ASF species are described elsewhere [18]. Quantitative polymerase chain reaction (qPCR) was performed using the ABI Prism 7700 with the SYBR green reporter [18]. Standard curves for measuring copy numbers of the 16S rDNA for the respective ASFs were generated using serial dilutions of linerized plasmid DNA containing each of 8 ASF bacteria 16S rDNAs as described previously [18], The quantities of each target ASF DNA were expressed as copy numbers of ASF 16S DNA per µg mouse DNA which was determined using the 18S rDNA-based primers and probe (Applied Biosystems).

### Gallbladder mRNA and cDNA Preparation and Gene Expression Analysis

Gallbladder RNA was extracted using the All-Prep DNA/RNA Micro Kit (Qiagen, Valencia, CA) and cDNA was prepared using the High Capacity cDNA Reverse Transcription Kit. Quantitative PCR was performed in the ABI Prism 7700 sequence detection system with SYBR green reporter and GAPDH was used as a housekeeping gene for comparative analysis. Fold changes in gene expression between the two groups (n = 5 per group) were determined using the ΔΔC_T_ method.

### Statistical Analyses

Prism (Graphpad, San Diego, CA) was used to perform statistical analyses. Data were analyzed by either One-Way ANOVA with Tukey’s post-test (Normalized gallbladder weight, and cecal colonization of ASF species), t-test (lipid analysis, and mucin gene expression). Mann-Whitney U test was utilized for data collected through scoring systems (histopathological scoring and mucin gel score). Prevalence data were analyzed using contingency tables and Fisher’s exact test. A P value of less than or equal to 0.05 was considered significant for all studies. Graphs are presented as mean and standard error calculated using the same statistical software.

## Results

### BALB/c Substrain Experiments to Determine Genetic Susceptibility

**Figure 1:**
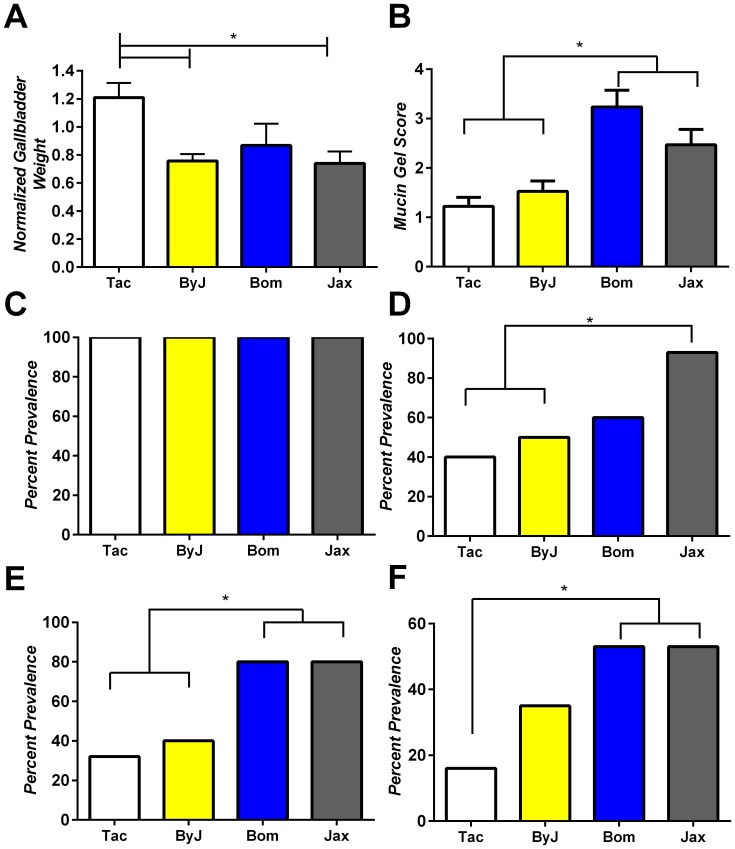
Biliary phenotype of genetically related BALB/cAnNTac (Tac) and BALB/cByJ (ByJ) and BALB/cJBomTac (Bom) and BALB/cJ (Jax) mice. (A) Normalized gallbladder weight (B) mucin gel score (C) Liquid crystal phase separation (D) cholesterol monohydrate crystal formation, (E) sandy stone formation and (F) cholesterol gallstone formation were analyzed after 8 weeks of lithogenic diet feeding. (A) Normalized gallbladder weight significantly differed between Tac mice and both ByJ and Jackson mice (*P<0.05). (B) Mucin gel score for both BALB/cJ and BALB/cJBomTac mice was significantly elevated compared to either Tac or ByJ mice (P<0.05). (C) Liquid crystal phase separation occurred in all animals studied. (D) Cholesterol monohydrate crystal formation occurred significantly more in BALB/cJ mice compared to both ByJ and Tac mice (*P<0.05). (E) Both BALB/cJ and BALB/cJBomTac mice compared to either Tac or ByJ mice displayed significantly increased sandy stone formation (*P<0.05). (F) Cholesterol gallstone formation was significantly increased in BALB/cJ and BALB/cJBomTac mice compared to Tac mice (*P<0.05).

To initially characterize the various substrains of BALB/c mice an 8 week lithogenic diet feeding study was conducted and gallstone phenotype was examined. Normalized gallbladder weight was significantly increased in BALB/cAnNTac mice (1.2+/−0.1 mg/g) compared to either BALB/cJ or BALB/cByJ mice (0.74+/−0.1 mg/g and 0.75+/−0.05 respectively; *P*<.05; Figure 1A). BALB/cJ mice (2.47+/−0.3) and BALB/cJBomTac mice (3.23+/−0.3) demonstrated significantly increased mucin gel scores in comparison to both BALB/cAnNTac and BALB/cByJ mice (1.2+/−0.2 and 1.53+/−0.2; P≤0.05; Figure 1B). All groups displayed the presence of phospholipid/cholesterol liquid crystals indicative of phase separation from supersaturated bile (Figure 1C). Significantly more BALBc/J mice (93%) developed cholesterol monohydrate crystals compared to BALB/cAnNTac and BALB/cByJ mice (P<0.05). The percent prevalence of sandy stones were significantly increased for both BALB/cJ and BALB/cJBomTac mice (80%) when compared to both BALB/cAnNTac mice (32%) and BALB/cByJ mice (40%) (*P*<0.05; Figure 1E). Finally, the percent prevalence of cholesterol gallstones was significantly increased for both BALB/cJ and BALB/cJBomTac mice when compared to BALB/cAnNTac mice (*P<0.05; Figure 1F). The prevalence differences noted for BALBc/J and BALBc/AnNTac are consistent with those described in our previous publication [6].

**Figure 2:**
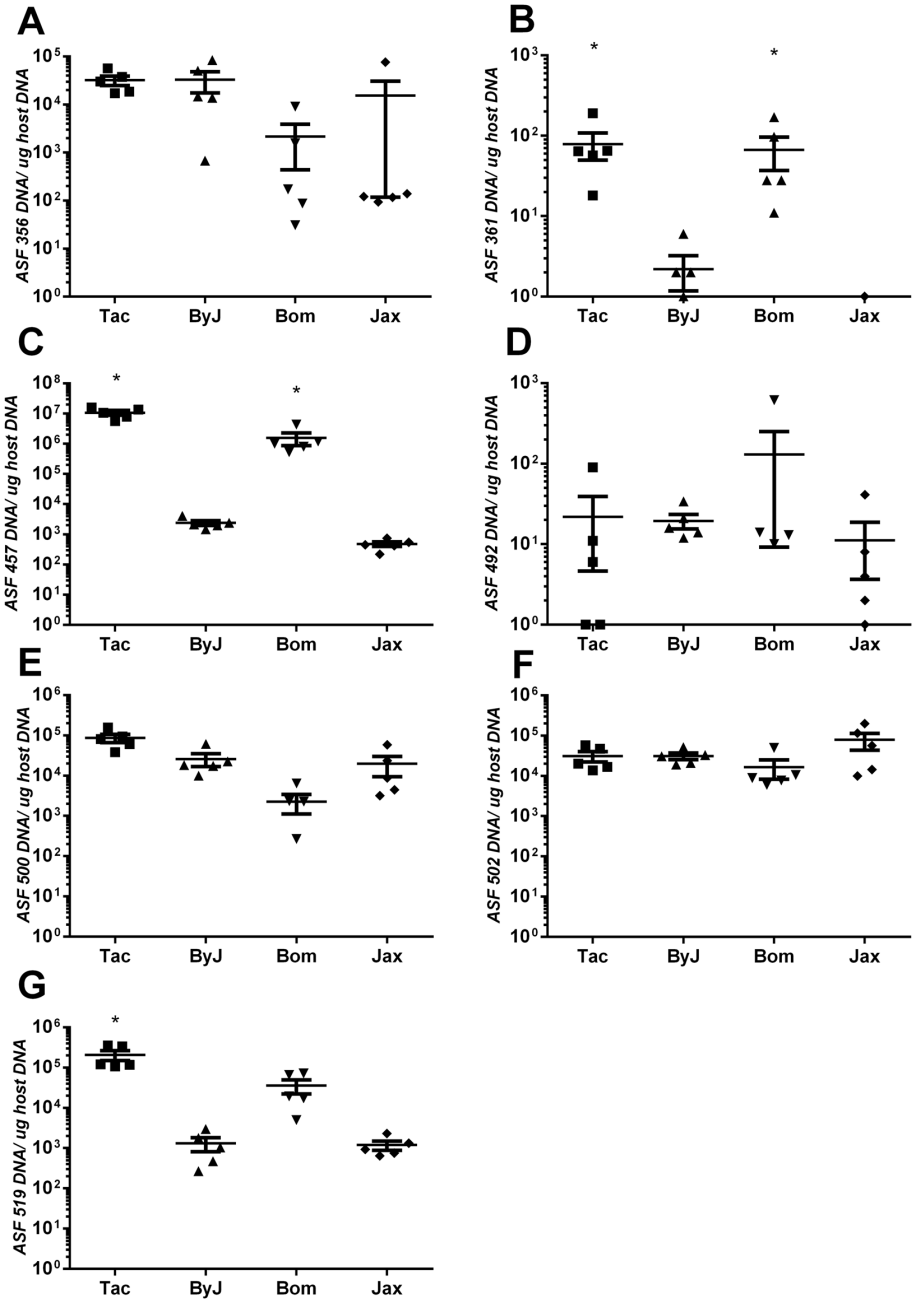
Microbial colonization of 8 ASF species in the ceca of mice from different vendors. (A) ASF 356, (B) ASF 361, (C) ASF 457, (D) ASF 492, (E) ASF 500, (F) ASF 502 and (G) ASF 519 were all evaluated by quantitative PCR and normalized to host tissue levels. Both ASF 361 (B) and ASF 457 (C) were significantly (*P<0.05) higher in mice from Taconic compared to Jackson origin mice. ASF 519 was significantly (*P<0.05) elevated in mice from one Taconic source (Tac) but not from the other Taconic source (Bom) (G). All other ASF species were colonized similarly among mice from both Jackson and Taconic sources.

Mice of Taconic origin displayed significantly increased colonization with ASF 361 and 457 compared to mice from Jackson confirming microbial differences (P<0.05; Fig. 2A–C). ASF 360 was not readily detected (data not displayed), ASF 519 was elevated in Tac but not BALB/cJBomTac strains of mice and all other ASF species demonstrated similar colonization levels between substrains of mice and were not analyzed further in subsequent studies (Fig. 2). These data indicate that different aspects of cholelithogenesis appear to be influenced both by vendor relatedness and genetic relatedness.

### Crossfaunation and Modification of Indigenous Gastrointestinal Microbiota in Adult BALB/c Substrains: Prevalence of Cholesterol Gallstones is not Significantly Altered

**Figure 3:**
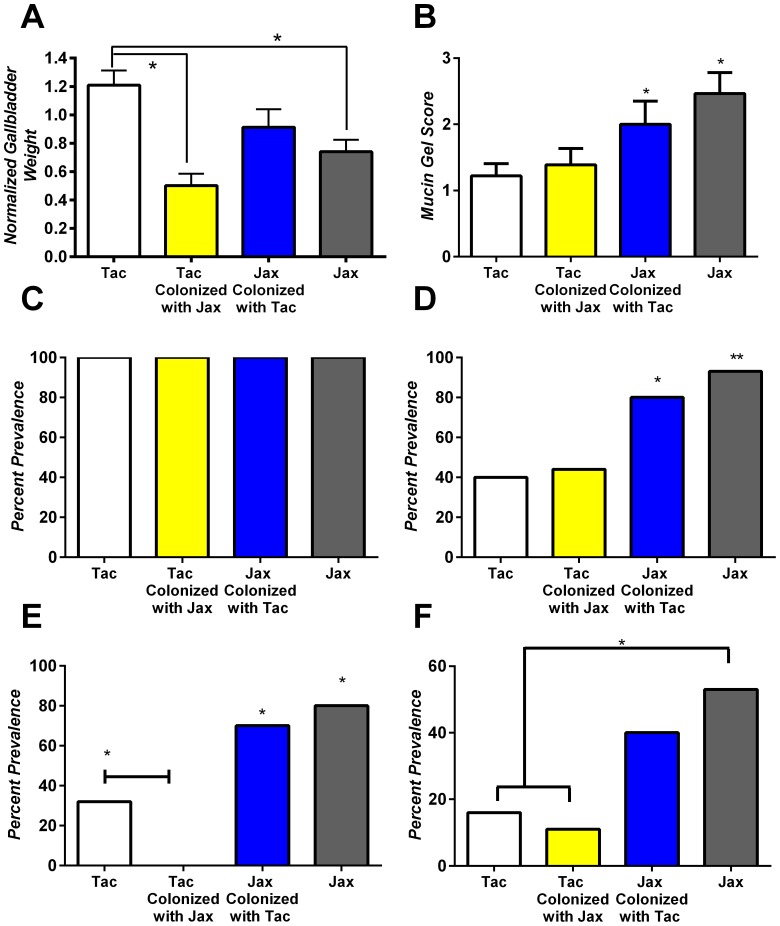
Components of the biliary phenotype may be altered by transferring flora to adult mice. (A) Normalized gallbladder weight (B) mucin gel score (C) Liquid crystal phase separation (D) cholesterol monohydrate crystal formation, (E) sandy stone formation and (F) cholesterol gallstone formation were analyzed after 8 weeks of lithogenic diet feeding. (A) Both Jax and Tac mice colonized with Jax flora developed significantly smaller gallbladders when compared to Tac mice (*P<0.05) (B) Jackson origin mice regardless of flora produced higher mucin scores compared to control Taconic mice (*P<0.05). (D) Flora transferred Jax mice displayed significant increases in cholesterol monohydrate crystal formation compared to control Tac mice (*P<0.05) and control Jax mice displayed significant increases compared to both Taconic groups (**P<0.05). (E) Both groups of Jackson mice displayed significant increases in sandy stone formation compared to both groups of Taconic mice (P*<0.05). Control Taconic mice displayed significant increases in sandy stones when compared to Taconic mice that underwent crossfaunation (*P<0.05). (F) Jax mice displayed significant increases in cholesterol gallstone formation compared to both groups of Tac mice (*P<0.05).

To determine if modifying the indigenous gastrointestinal microbiota in adult mice with an already established microbiome could alter cholesterol gallstone prevalence BALB/cAnNTac mice were crossfaunated with feces from BALB/cJ mice and likewise in the reverse configuration. The normalized gallbladder weight significantly decreased in BABL/cAnNTac mice crossfaunated with BALB/cJ feces compared to unaltered BABL/cAnNTac mice (0.50+/−0.1 mg/g from 1.2+/−0.1 mg/g; *P*≤0.05; Figure 3*A*). Crossfaunating BALB/cJ mice with Taconic flora did not have a similar effect. With the exception of sandy stones formation, all other phenotypic parameters including mucin gel score and cholesterol gallstone formation remained unchanged statistically between crossfaunated groups of identical genetic backgrounds (Figure 3B–D, F). Taconic mice cross-faunated with Jax flora develop statistically fewer sandy stones when compared to control Taconic mice (Figure 3E; *P<0.05); however, the nearly identical prevalence rates of true cholesterol gallstones, a more advanced stage of cholelithogenesis, reduces the impact of this finding.

**Figure 4:**
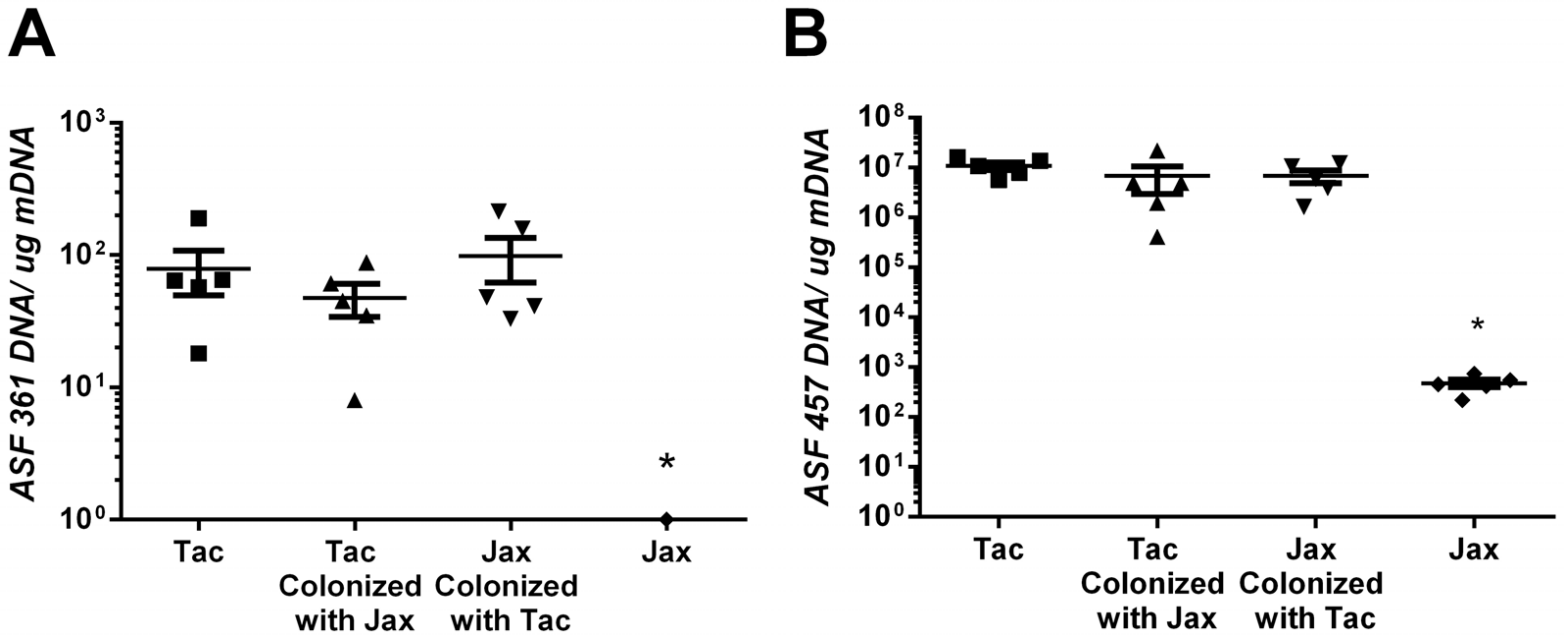
Transfer of Taconic flora to Jackson mice leads to significant increases in ASF457 (A) and 361. ASF361 (A) and 457 (B) were quantified from a cohort of cross-faunated mice (n = 5) by real-time PCR. There were significant elevations in the colonization of these two bacteria in mice that were of Taconic origin or those which received Taconic flora (*P<0.05). Colonization of Taconic mice with Jackson flora did not significantly diminish the colonization by these organisms.

Quantitative PCR indicated that Jackson mice given Taconic flora via oral gavage and continuous co-housing developed significantly greater colonization with ASF 457, and 361 (Figure 4). These results indicate that modulation of the microbiota of adult mice may alter some aspects of the cholesterol gallstone phenotype including gallbladder weight but does not appear to alter the formation of cholesterol gallstones.

### Crossfostering and Modification of the Indigenous Gastrointestinal Microbiota in Neonatal BALB/c Substrains does not Alter Significantly the Prevalence of Cholesterol Gallstones

**Figure 5:**
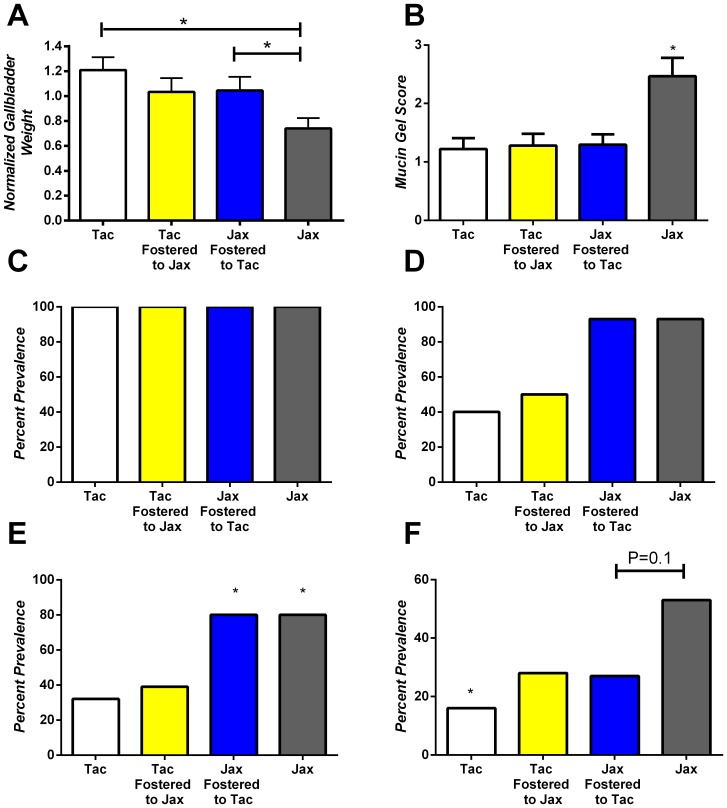
Components of the biliary phenotype may be altered by crossfostering mice shortly after birth. (A) Normalized gallbladder weight (B) mucin gel score (C) Liquid crystal phase separation (D) cholesterol monohydrate crystal formation, (E) sandy stone formation and (F) cholesterol gallstone formation were analyzed after 8 weeks of lithogenic diet feeding. (A) Both Tac and Jax fostered to Tac developed significantly larger gallbladders (*P<0.05) compared to Jax mice. (B) Jax mice displayed significant (*P<0.05) elevations in mucin gel accumulation compared to all other groups including Jax fostered to Tac. (D–E) Jax mice and Jax fostered to Tac developed significantly more (P<0.05) cholesterol monohydrate and sandy stones then either group from Taconic. (F) Tac mice developed significantly (P<0.05) less cholesterol gallstones then Jax mice and both fostered groups displayed intermediate and non-significantly different levels of cholesterol gallstone formation.

**Figure 6:**
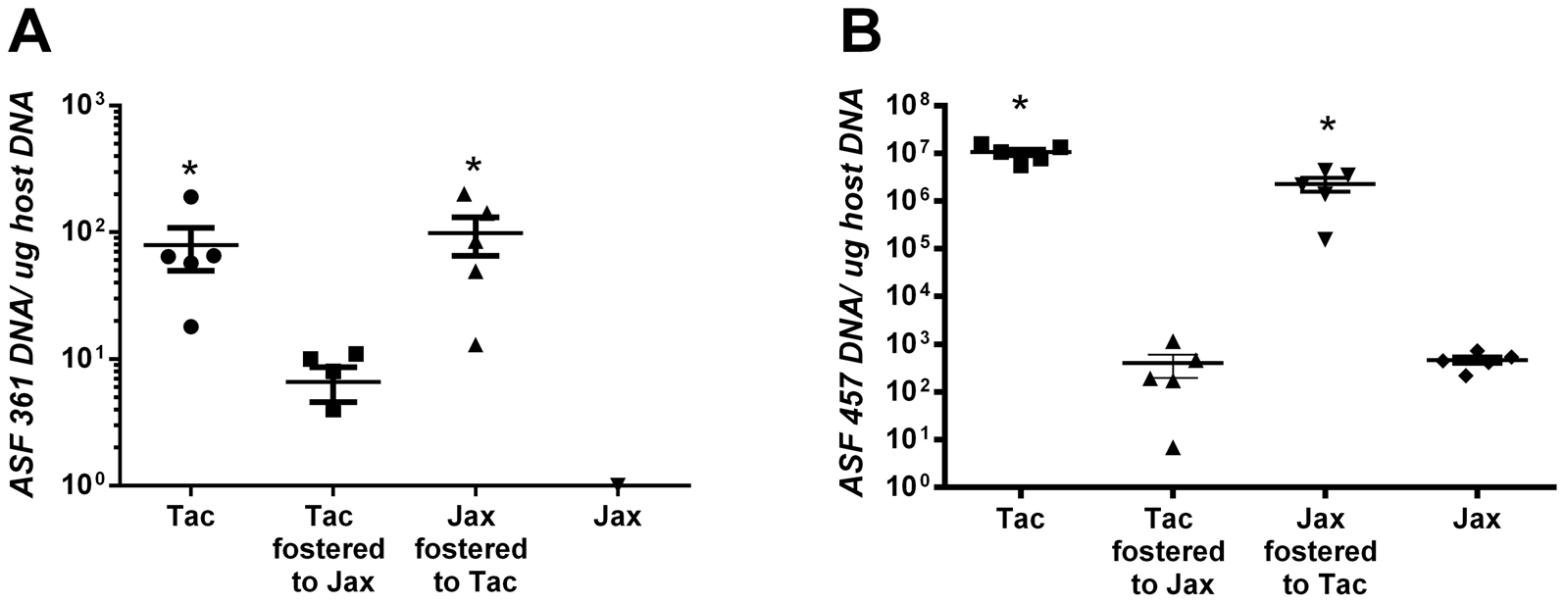
Crossfostering of mice leads to significant alterations in ASF361 and 457. ASF361 (A) and 457 (B) were quantified from a cohort (n = 5) of cross-fostered mice by real-time PCR. There were significant elevations in the colonization of these two bacteria in mice of Jackson origin crossfostered to Taconic mothers and significant decreases in Taconic pups crossfostered to Jackson mothers (*P<0.05) indicating effective alteration of the flora.

Newborn BALB/cAnNTac pups were crossfostered to BALB/cJ dams and newborn BALB/cJ mice were cross-fostered to BALB/cAnNTac mothers to determine if modifying the indigenous gastrointestinal microbiota of the mice prior to the establishment of resident flora could alter cholesterol gallstone phenotype. The normalized gallbladder weight of Jackson mice fostered to Taconic mice increased significantly compared to their unfostered counterparts (P≤0.05; 1.05+/−0.1 mg/g and 0.74+/−0.08 mg/g respectively) (Figure 5A). Crossfostering Jackson mice to Taconic mice also significantly decreased the mucin gel score when compared to their unfostered counterparts (1.29+/−0.2 and 2.46+/−0.3 respectively; *P*≤0.05; Figure 5B). Crossfostering of either group failed to significantly alter any other cholesterol gallstone parameter (Figure 5D–F); however, there was a large, decrease in the prevalence of cholesterol gallstones in Jackson mice fostered to Taconic mothers (P = 0.1 Figure 5F). Quantitative PCR of ASF 457 and 361 (Figure 6) confirmed an alteration of the microbiome in both crossfostered groups. Altering the gastrointestinal microbiome at an early age alters some of the cholesterol gallstone phenotype of mice including mucin gel accumulation and gallbladder size, but does not significantly alter the prevalence of cholesterol gallstones.

### Germ-free Mice Demonstrate Augmented Cholesterol Gallstone Pathogenesis

**Figure 7:**
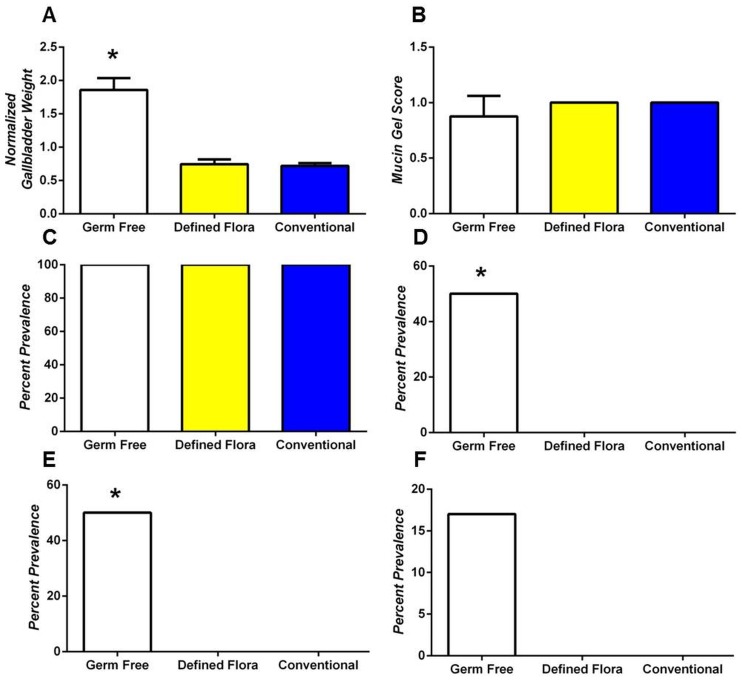
Germ-free mice are more susceptible to cholesterol cholelithogenesis (A) Normalized gallbladder weight (B) mucin gel score (C) Liquid crystal phase separation (D) cholesterol monohydrate crystal formation, (E) sandy stone formation and (F) cholesterol gallstone formation were analyzed after 8 weeks of lithogenic diet feeding. (A) Germ-free mice developed significantly increased normalized gallbladder weights compared to either conventional or ASF colonized mice (*P<0.05). Mucin gel score did not significantly differ among groups (all conventional and ASF mice developed mucin gel scores of “1”). (D–E) Germ-free mice developed significantly more cholesterol monohydrate (D, *P<0.05) and Sandy stones (E, *P<0.05) and had an non-significant increase in cholesterol gallstones (F).

Germ-free mice fed the lithogenic diet displayed significant increases in normalized gallbladder weight (Figure 7A), cholesterol monohydrate crystal formation (Figure 7D) and sandy stone formation (Figure 7E) when compared to either conventionally housed mice or mice colonized with a defined flora (*P<0.05). Mucin gel score (Figure 7B), formation of liquid crystals (Figure 7C) and formation of true cholesterol gallstones (Figure 7F) did not differ statistically among the latter two groups; however, germ-free mice had an increase in cholesterol gallstones consistent with the statistically significant increased amount of sandy stones that formed.

**Figure 8:**
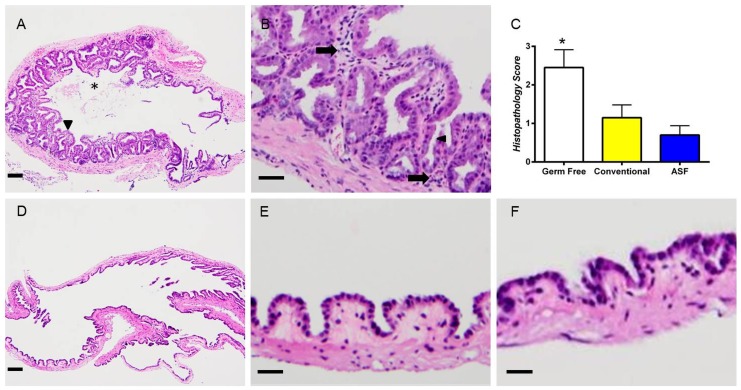
Germ-free mice develop significantly more severe gallbladder histopathological changes compared to ASF colonized mice. (A, B) Low power and high power image of gallbladders from Germ-free mice demonstrate hyperplasia (arrowhead), inflammation (arrow) and the presence of eosinophilic luminal material which is consistent with mucin gel (asterisk). (C) Overall, gallbladder histopathology score which examines inflammation, edema, and hyperplasia was significantly increased (*P<0.05) in Germ-free mice compared to ASF colonized mice and nearly significant (P = 0.07) when compared to conventional mice. Both ASF colonized (D, low power, E, high power) and conventional (F, high power) mice display minimal inflammation and maintain a normal epithelial lining of a single layer of cuboidal epithelium.

Germ-free mice displayed moderate hyperplasia of the gallbladder epithelium (arrowhead Figure 8A and B), accumulation of inflammatory infiltrates (arrow; figure 8 A and B) and luminal deposition of eosinophlic material consistent with mucin accumulation (asterisk; Figure 8A). Germ-free mice displayed significant increases in gallbladder histopathology score when compared to either conventional or ASF colonized mice (Figure 8C) and gallbladders from ASF colonized (Figure 8 D, E) and conventional mice (Figure 8F) displayed normal gallbladder epithelium consisting of a single cuboidal layer and minimal inflammatory infiltrates.

### Germ-free Mice Exhibit Increased Expression of Gallbladder Mucin Genes

**Figure 9:**
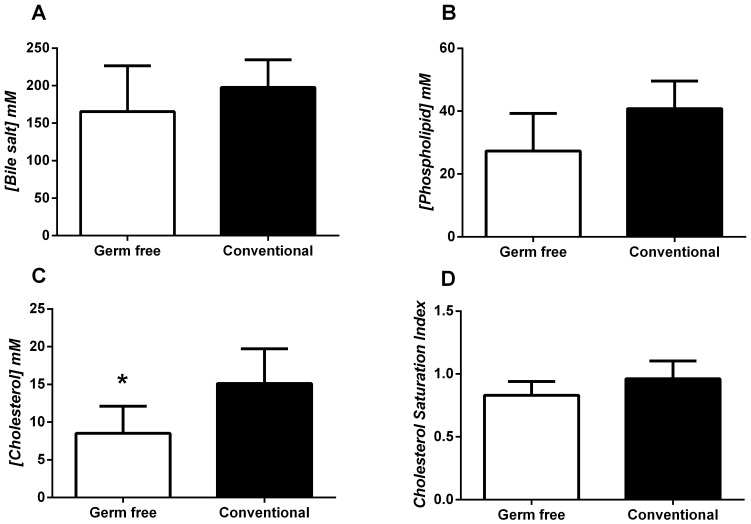
Germ-free mice do not exhibit a significantly increased gallbladder cholesterol saturation index. (A, B) The concentration of gallbladder bile salts and phospholipids was not significantly altered between conventionally reared-mice and germ-free mice. (C) Gallbladder cholesterol concentration is mildly yet significantly decreased in germ-free mice compared to conventionally reared mice (*P*<0.05). (D) Overall the gallbladder CSI did not differ significantly between germ-free and conventionally reared mice.

Despite displaying similar mucin gel scores histopathological analysis demonstrated the presence of what appeared to be mucin accumulation in germ-free but not ASF colonized or conventionally reared mice. To further analyze this we performed quantitative PCR on mucin genes of the gallbladder. Compared to conventional mice, germ-free mice display significant upregulation of *Muc1*, (Figure 9A) *Muc3* (Figure 9B), and *Muc4* (Figure 9C) genes (*P<0.05). In contrast, *Muc5ac* (Figure 9D) and *Muc5b* (Figure 9E) remained relatively unchanged. These results indicate that despite similar mucin gel scores, mucin gene expression in these groups is changed significantly.

### Germ-free Mice Produce a Similar Gallbladder Bile Cholesterol Saturation Index to Conventionally Reared Mice

**Figure 10:**
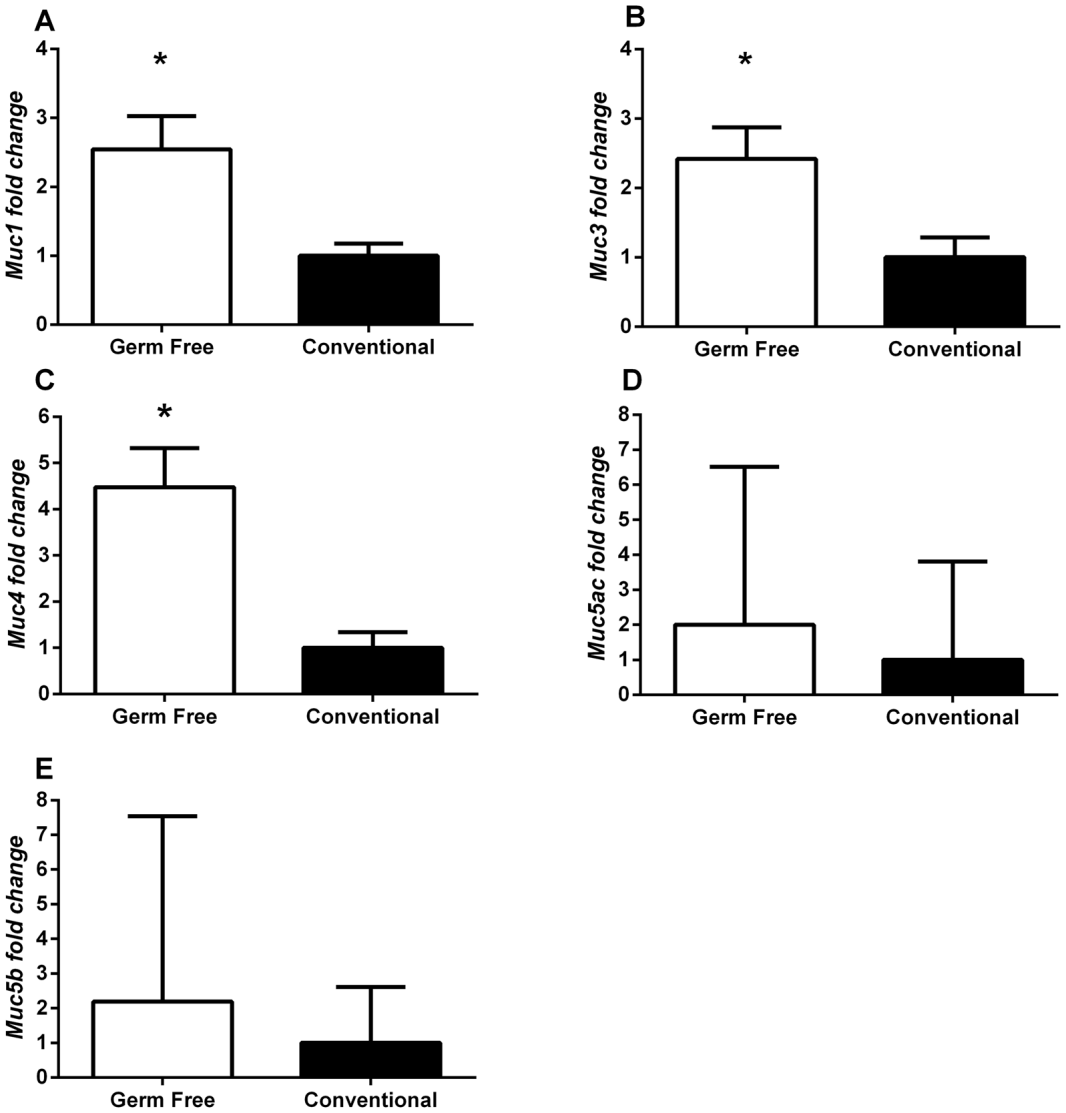
Mucin gene expression is significantly increased in germ-free mice compared to conventionally reared mice. (A) *Muc1*, (B) *Muc3*, and (C) *Muc4* are all significantly (*P*<0.05) increased (∼2.5–4.5 fold) in germ-free mice when compared to conventionally reared mice. Neither (D) *Muc5ac* nor (E) *Muc5b* displayed any differences and expression was highly variable among mice.

The indigenous microbiota has been previously demonstrated to alter a variety of metabolic pathways [22]–[24]. Because of this we wished to determine if altered relative lipid concentration of gallbladder bile was contributing to the differences in cholesterol gallstone phenotype noted in these mice. Neither bile salt concentration (Figure 10A), nor phospholipid concentration (Figure 10B) differed significantly among germ-free and conventionally housed mice; however, bile cholesterol concentration was slightly, yet significantly lower in germ-free mice compared to conventionally reared mice (*P<0.05; Fig. 10C). This alteration in cholesterol concentration did not alter the cholesterol saturation index of the gallbladder bile in these groups of mice (Figure 10 D).

## Discussion

The purpose of this study was to investigate the interacting roles of the indigenous gastrointestinal microbiota and genetic differences in cholesterol gallstone formation in closely related inbred substrains of mice. We began this study by analyzing mice housed under SPF conditions. Initial analysis revealed that cholelithogenesis appeared to be dependent on both genetic relatedness and vendor origin. For example, genetically related BALB/cJ and BALB/cJBomTac mice both displayed marked significant increases in mucin gel accumulation and sandy stone formation compared to genetically related ByJ and Tac mice. In contrast, Tac and ByJ displayed significant differences in normalized gallbladder weight. Further analysis utilizing procedures to alter the indigenous gastrointestinal microbiome of SPF housed mice indicated that alterations in the microbiome altered aspects of the cholesterol gallstone phenotype, most notably gallbladder size and mucin content. Finally, when genetically identical mice were re-derived into a germ-free state they became more susceptible to cholesterol cholelithogenesis when compared to their counterparts which either were housed in SPF conditions or colonized only with ASF. Germ-free mice demonstrated significantly increased gallbladder size consistent with altered gallbladder motility, increased gallbladder inflammation and increased mucin gene expression. These data are consistent with a variety of studies in inbred and recombinant inbred strains and humans which demonstrate the overlapping influences of environmental and genetic factors in cholesterol gallstone pathogenesis [5], [25], [26].

Cross-fostering and crossfaunation studies provided evidence that altering the indigenous intestinal microbiome might impact cholesterol gallstone pathogenesis however there were limitations in these initial studies. Specifically, despite these substrains of mice being closely related they are not genetically identical so microbial alterations still occurred in the presence of varied host genetics. Also, we examined only the eight ASF species described and did so only in the cecum of mice. Since Taconic mice will invariably develop a more complex flora, and the ASF organisms preferentially colonize a variety of locations in the gastrointestinal tract cecal PCR will not detect all microbial similarities or differences [10], [18]. Likewise, fecal slurries used for crossfaunation studies were not microbiologically characterized. A final complicating factor in cross-fostering studies is that some agents may be transferred from the mother to her pups prior to cross-fostering. Due to these potentially complicating factors, we carried out additional follow-up studies in germ-free mice and compare these to conventionally housed SPF mice as well as germ-free mice colonized with ASF. These additional studies supported our initial data insofar as they demonstrated the relative contribution of gastrointestinal microbes to gallbladder size, a surrogate marker of gallbladder motility. Further, these studies supported the role of the indigenous microbiome in gallbladder mucin accumulation because there were significant increases in mucin gene expression in germ-free mice. These alterations in mucin gene expression occurred without any significant difference in mucin gel-score; however, this is likely the result of differences in sensitivity between these two assays and accumulation of mucin was noted on histopathological sections of gallbladders from germ-free mice supporting the gene expression changes noted. An additional finding in germ-free studies was that the germ-free mice demonstrated significant increases in histopathological damage to the gallbladder mucosa. Notably, these mice demonstrated hyperplasia, and inflammation and edema of the gallbladder. These findings are consistent with our previous studies which demonstrated a role for inflammation in gallstone formation in BALB/c substrains of mice [6].

In all of our studies, alteration of the microbiome led to an alteration of normalized gallbladder weight (either up or down depending on the transfer). The primary factors involved in gallbladder emptying and filling respectively are cholecystokinin and fibroblast growth factor 15 [27], [28]. Both of these factors are produced as hormones in the intestinal tract where the transferred bacteria colonize. Interestingly, cholecystokinin secretion is influenced by innate immune stimulation implying that the indigenous microbiome may alter gallbladder volume by this mechanism [29].

Mucin gel accumulation was significantly decreased when Jackson mice were fostered to Taconic mice and mucin gene expression was significantly elevated in germ-free mice. Since mucin gel accumulation is associated with gallstone formation and appears to be the predominant nucleation matrix, this influences gallstone prevalence [30]. The interaction between the indigenous microbiome, the immune system and inflammation is becoming well established [31]. Likewise, mucin genes are known to be regulated by inflammatory molecules making it reasonable to hypothesize that the microbiome alters mucin gene expression through immunomodulation [32]–[36]. Consistent with this hypothesis, germ-free mice display significant increases in immunohistopathology of the gallbladder.

Biliary cholesterol saturation index (CSI) was not altered significantly between germ-free and SPF mice indicating that the increased rate of cholesterol gallstone pathogenesis in germ-free mice was not due to increased gallbladder bile CSI. In fact, the data demonstrate that the overall concentration of gallbladder cholesterol is significantly decreased in germ-free mice. This decrease in cholesterol concentration may be due to increased *Muc1* expression because *Muc1* induction has been shown to increase cholesterol absorption from gallbladder bile which in turn has been shown to promote gallbladder inflammation and hypomotility [37], [38].

The overall gallstone prevalence in germ-free studies was reduced compared to our SPF studies. This finding is likely related to differences in the diet utilized and handling of the diet. Initial studies utilized a diet procured directly from The Jackson laboratory. This diet has been utilized extensively by our group in the past. Because this diet was not sterile nor was it packaged in a fashion consistent with maintenance of sterility for the germ-free studies we needed to utilize an alternative diet. This diet was formulated at a different location, and utilized slight variations in the source of lipids and was irradiated. It is possible that such changes might have contributed to the altered gallstone prevalence rates noted. Nevertheless, in direct comparison, germ-free mice fed this diet experienced significant increases in cholesterol gallstone pathogenesis (increased cholesterol monohydrate crystal and sandy stone formation) when compared to mice colonized with ASF or those maintained conventionally.

Taken together our data indicate that alterations in the intestinal microbiome may alter cholesterol gallstone pathogenesis in a manner consistent with alteration of gallbladder motility, localized inflammation, increased mucin gene expression and mucin gel accumulation. These findings are consistent with an increasing body of literature which describes the role of the microbiome in a variety of immunological and metabolic diseases [12], [31], [39]–[41]. Microbial characterization of human populations at risk for cholesterol gallstone formation may serve as a valuable tool for predicting individuals that may be at greater risk for cholesterol gallstones.

## Supporting Information

Figure S1 Relatedness of the strains examined with regard to genetics and vendor.
(TIF)

Figure S2 A schematic diagram depicting how cross-faunation studies were conducted. Feces was gavaged to mice and then recipient animals were co-housed with donors to ensure continued microbial exposure.
(TIF)

Figure S3 A schematic diagram depicting how cross-fostering studies were conducted. Mice were monitored every morning and when pups were first noted they were immediately transferred to recipient mothers of the opposing strain. These mice were then used as founders for generation of mice for subsequent studies.
(TIF)

Figure S4 Colonization of germ-free mice with ASF species was confirmed by quantitative PCR of cecal tissue. Colonization of germ-free mice with 7 of the 8 ASF species was detected reliably by cecal QPCR. Low levels of ASF 360 were found in these mice consistent with mice from Taconic previously analyzed.
(TIF)
