# Supplementary figures and images for: An Analysis of the Role of the Indigenous Microbiota in Cholesterol Gallstone Pathogenesis
Source: PLoS One. 2013 Jul 29;8(7):e70657. doi: 10.1371/journal.pone.0070657 (PMC3726617; doi:10.1371/journal.pone.0070657)

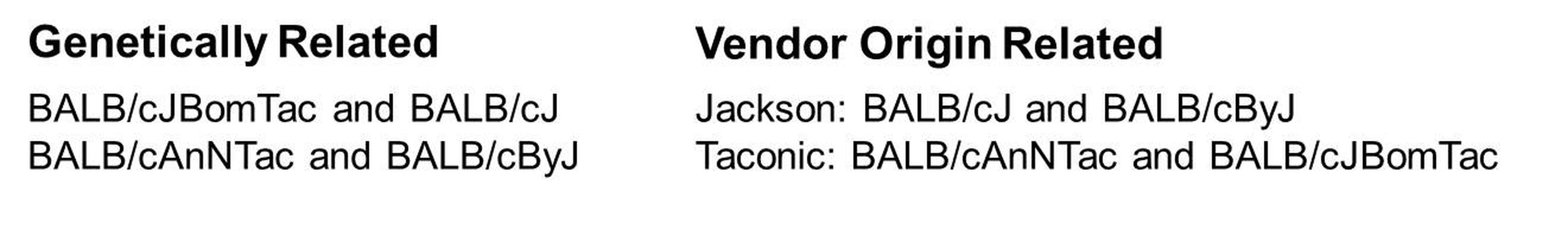

Supplement: Figure S1 — Relatedness of the strains examined with regard to genetics and vendor. (TIF) [file pone.0070657.s001.tif]

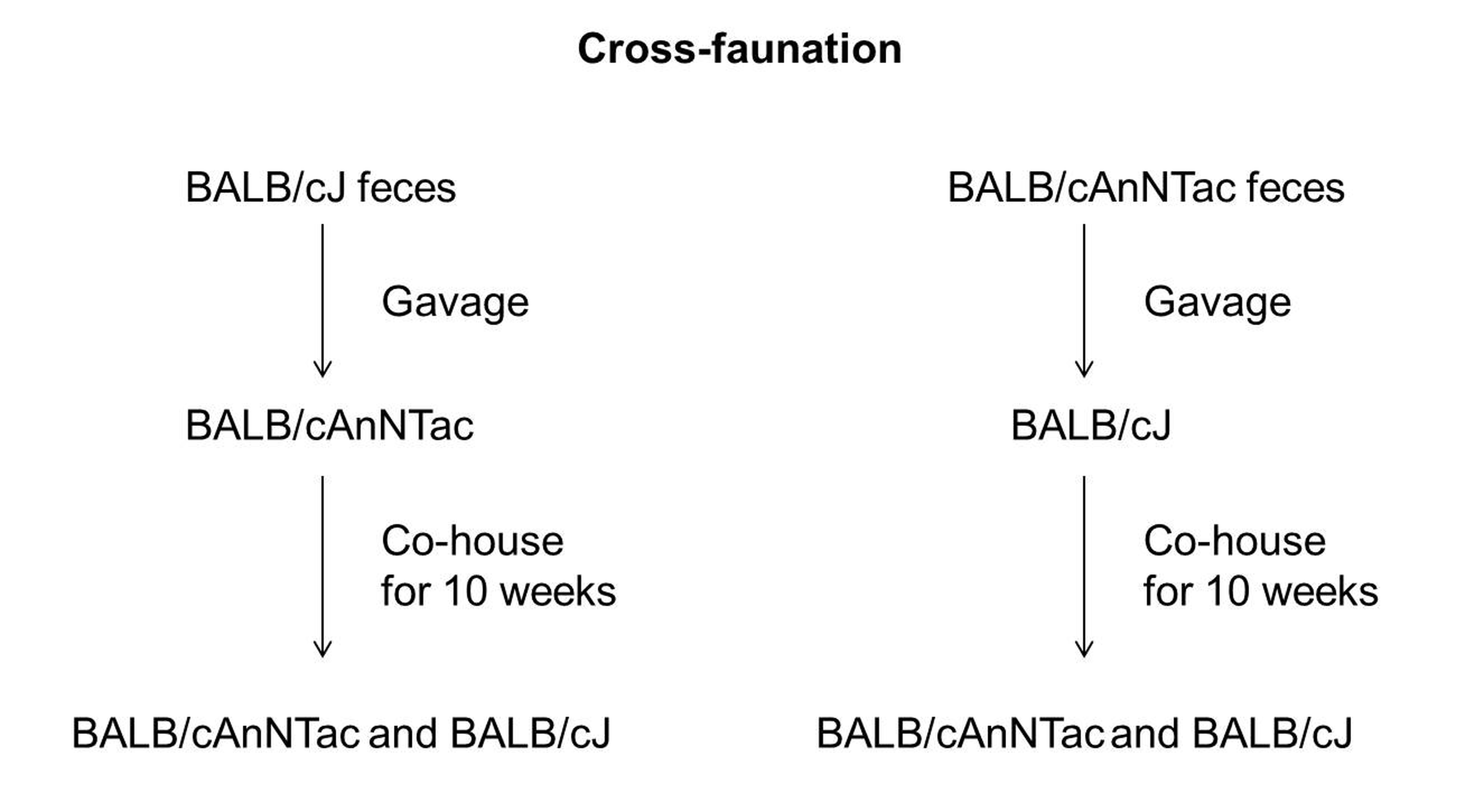

Supplement: Figure S2 — A schematic diagram depicting how cross-faunation studies were conducted. Feces was gavaged to mice and then recipient animals were co-housed with donors to ensure continued microbial exposure. (TIF) [file pone.0070657.s002.tif]

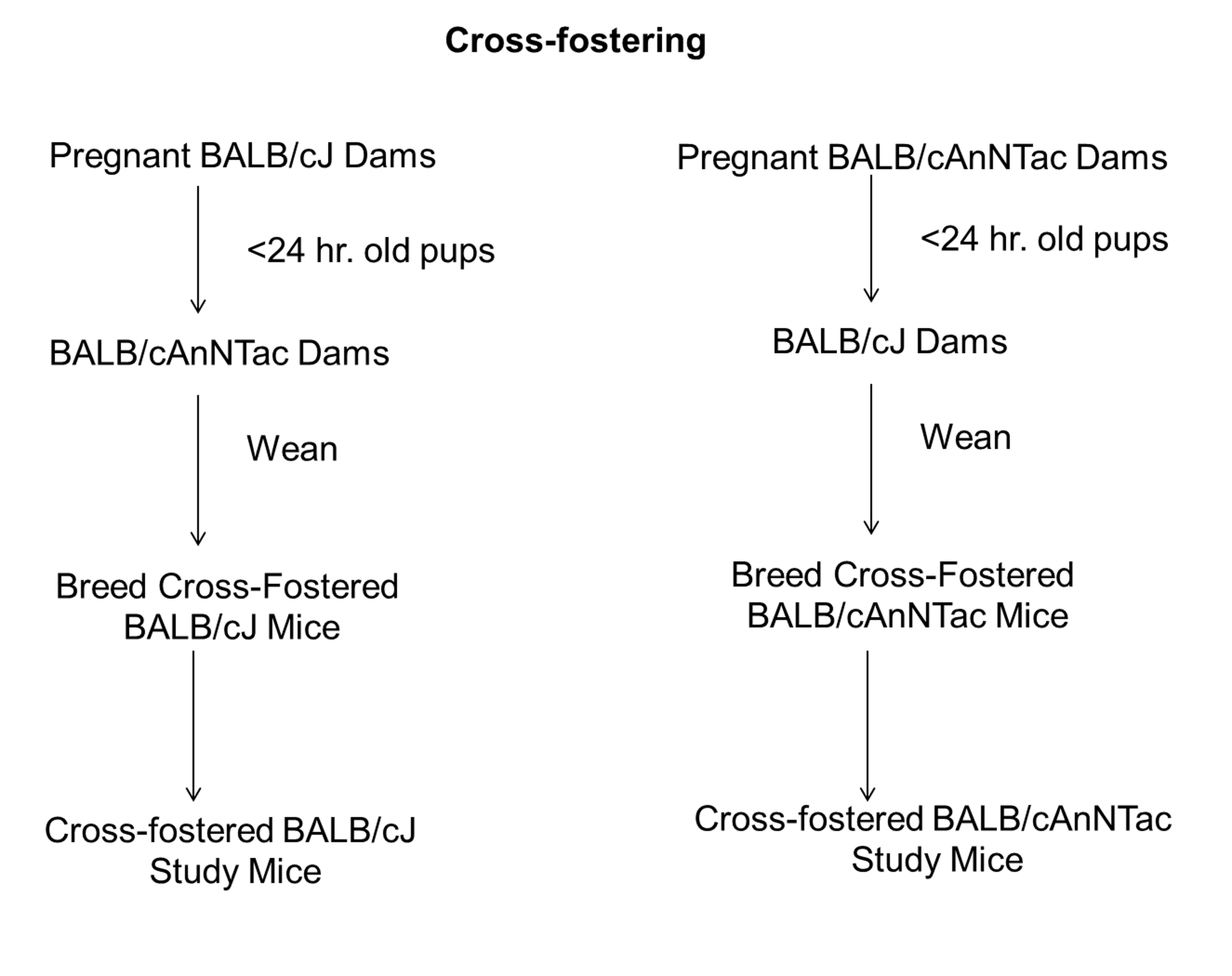

Supplement: Figure S3 — A schematic diagram depicting how cross-fostering studies were conducted. Mice were monitored every morning and when pups were first noted they were immediately transferred to recipient mothers of the opposing strain. These mice were then used as founders for generation of mice for subsequent studies. (TIF) [file pone.0070657.s003.tif]

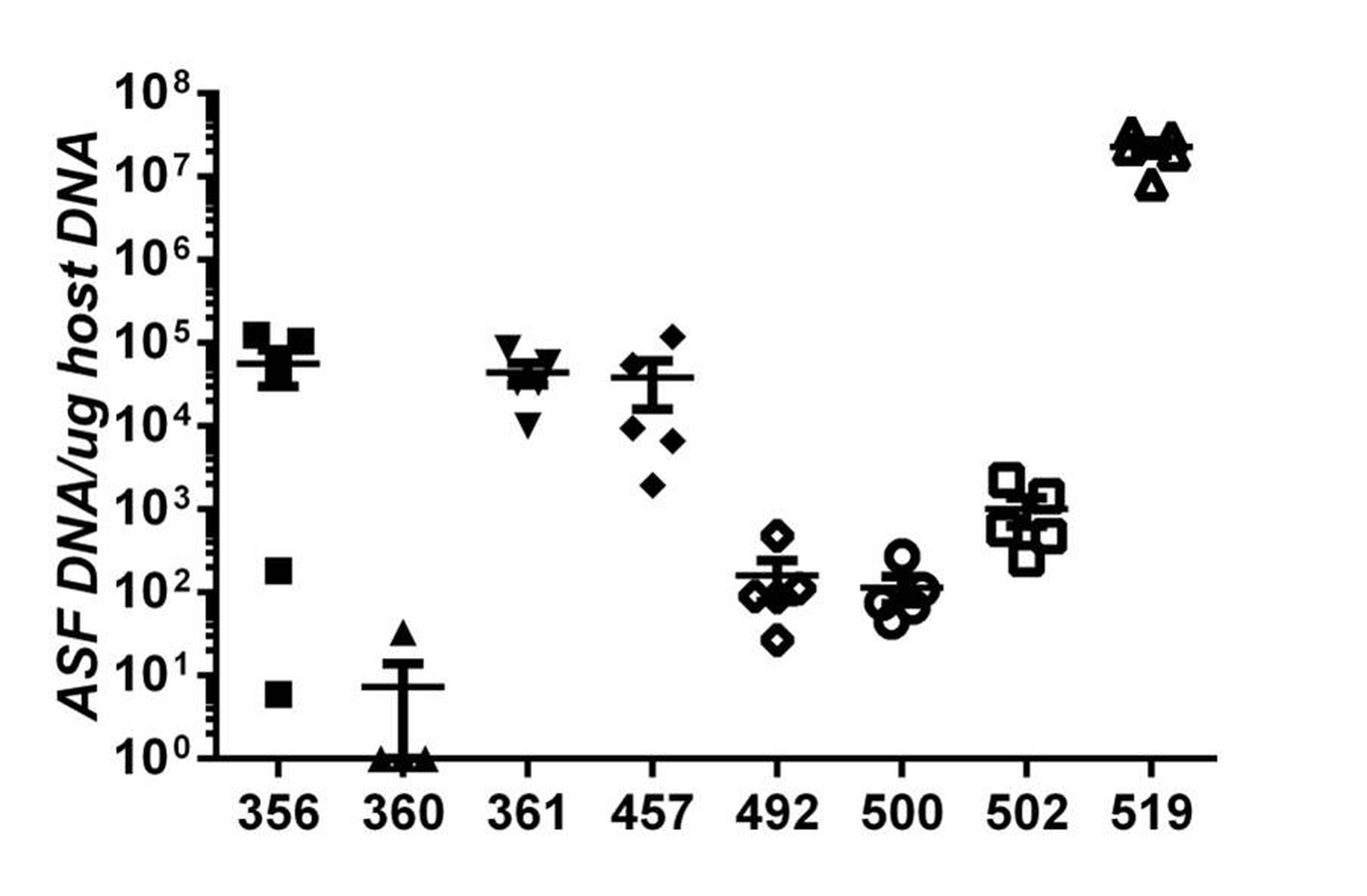

Supplement: Figure S4 — Colonization of germ-free mice with ASF species was confirmed by quantitative PCR of cecal tissue. Colonization of germ-free mice with 7 of the 8 ASF species was detected reliably by cecal QPCR. Low levels of ASF 360 were found in these mice consistent with mice from Taconic previously analyzed. (TIF) [file pone.0070657.s004.tif]
